# Clinical Remarks upon Surgical Cases in the Buffalo General Hospital—Caries of the Humerus—Artificial Pupil—Glandular Tumor Removed from Superior Carotid Triangle

**Published:** 1869-01

**Authors:** J. F. Miner


ART. IV.—*Clinical Remarks upon Surgical Cases in the Buffalo General Hospital—Caries of the Humerus—Artificial Pupil—Glandular Tumor removed from Superior Carotid Triangle.* BY J. F. MINER.

1*st.* *Caries of Bone.*—This young man, now 16 years old, suffers from what appears to be caries of the upper portion of the humerus. This is indicated by the small openings you observe, which give exit to a constant discharge of pus, by pain, redness and swelling, and above all, by the passage of the probe down upon denuded bone, that is, bone which has no periosteal covering. This condition of disease is of about four months' continuance, and he urges operative interference very strongly, though his malady is, as yet, of comparatively short duration. Caries of bone is analogous to ulceration in the soft parts. Children are most liable to be affected by it, and it is most frequently situated in the soft bones, or in the articular portions of the long bones, where the areolar structure predominates over the compact tissue. The causes of this disease are not fully known, and it often appears where no adequate cause can be traced, as is the case in the instance before you. The causes are divided into local and general; the latter of which are believed to be the more common and potent. Of the first, all direct injuries, by which bone is denuded of its periosteal covering, and all concussions which in any way interrupt its nutrition, may be the cause of such disease. Of the constitutional causes, all influences which induce debility, and impair the general health, may be included, mercury, rheumatism, scurvy, syphilis and scrofula, to which are added all the acute diseases which often end in great impairment of the system and health. The influence of these causes appears probable, but the connection is certainly oftentimes remote and very uncertain. In the absence of known cause scrofula receives the credit in too many instances. If scrofula is as common cause as is generally supposed, the natural history of caries occurring in young persons must be re-written. Scrofula, or that condition of system which leads to tubercular deposits in later life has very little natural tendency to recovery, after manifesting itself by such obvious organic disease, and if we go still farther and adopt the idea that caries of bone is of tubercular character, we must at once conclude that its natural tendency and termination is progressively destructive. This view appears to me wholly inconsistent with daily experience; these cases have a well-marked and constant tendency to recovery, and under favorable circumstances the great majority of this and similar affections, do progress to a favorable termination, showing conclusively, that at least, they are not tubercular.

I believe this young man would recover if left to himself, and the main object in opening down upon the bone and removing this ulcerated or carious part, is to assist and expedite the process of nature, imitating her example and following her guidance. These operations for removal of diseased bone are rarely followed by much local or general disturbance, so that if we fail to do great good we have no reason to fear much harm.

2*d*. *Artificial Pupil.*—Operation for artificial pupil offers to this patient his only chances of restored vision. The eye has been for a long time the seat of inflammatory disease, and our opportunities of determining his prospects of vision are lost so far as examination of the retina and choroid are concerned. The pupil has been closed by inflammatory products, and perhaps its margin glued to the capsule of the lens, constituting what is called *synechia posterior.* If so, the chances of success are greatly lessened, almost lost. The operation, as you observe, consists in making a small opening through the cornea, near the sclerotic, introducing a small, very fine hook, and drawing out a part of the iris, which is excised near to the cornea. The wound is carefully cleared of all of the iris, and treated as in operation for extraction of cataract. When both eyes are blind from closure of pupil this operation is indicated, but while useful vision is present in either eye, it should not be made, since nothing can thus be gained. Again, one eye only should be operated upon, and if good results are obtained nothing can be added to it by operation upon the second eye; from the impossibility of making artificial pupil so as to insure harmony in the two eyes, more embarrassment than benefit is liable to result.

Various methods have been proposed for making this operation, all of which will be described to you hereafter. The plan you have observed seems to me as feasible and safe as any, and may generally be adopted. It is remarkable how susceptible and ready to take on inflammation, the iris appears to be, if pressed upon by swollen or dislocated lens, or irritated by lodgment of foreign body, and yet with what safety it can be lacerated and excised. It is rare to observe inflammation of the iris after any of the operations made upon it for iridectomy or for artificial pupil.

*Glandular Tumor of the Neck.*—We are able to present for your examination what appears to be a glandular tumor, situated as you observe in the *superior carotid triangle*—one of the most important surgical regions. These growths are often located in the neck, and are supposed to be hypertrophied and otherwise diseased lymphatic glands. They are slow in growth, not usually painful, have a degree of elasticity, are generally irregular in outline, with lobulated surface. There can be little doubt of its character, encephaloid tumor being the only form of disease which it resembles. The history of the growth, the appearance of patient, and other conditions lead us to believe that it is not malignant. Upon the conviction that it is benign in character, that it does not involve the important arteries and nerves in this region, and that it can be safely removed, we open down freely upon its external surface, expecting to separate the diseased from the healthy tissues with the finger and with the handle of the scalpel rather than its edge. Such growths, though not inclosed in a proper sac or cyst, have yet a covering or artificial cyst made by their growth, condensing and pushing back the cellular tissue of the parts, so that a line of distinct separation can be traced; otherwise their removal by operation would be much more difficult, or even impracticable. Thus you observe this growth, weighing five or six ounces, has been safely removed from a deep, important, surgical region, where cutting at its base would be attended with the greatest difficulties and dangers.

